# A high-density electroencephalography study reveals abnormal sleep homeostasis in patients with rapid eye movement sleep behavior disorder

**DOI:** 10.1038/s41598-021-83980-w

**Published:** 2021-02-26

**Authors:** Amandine Valomon, Brady A. Riedner, Stephanie G. Jones, Keith P. Nakamura, Giulio Tononi, David T. Plante, Ruth M. Benca, Melanie Boly

**Affiliations:** 1 Psychiatry - Wisconsin Institute for Sleep and Consciousness, University of Wisconsin-Madison, 6001 Research Park Boulevard, Madison, WI 53719 USA; 2 School of Medicine and Public Health, University of Wisconsin-Madison, Madison, WI USA; 3 Neurology, University of Wisconsin-Madison, Madison, WI USA; 4 University of California Irvine, Psychiatry and Human Behavior, Irvine, CA USA

**Keywords:** Neurological disorders, Sleep

## Abstract

Rapid eye movement (REM) sleep behavior disorder (RBD) is characterized by disrupting motor enactments during REM sleep, but also cognitive impairments across several domains. In addition to REM sleep abnormalities, we hypothesized that RBD patients may also display EEG abnormalities during NREM sleep. We collected all-night recordings with 256-channel high-density EEG in nine RBD patients, predominantly early-onset medicated individuals, nine sex- and age- matched healthy controls, and nine additional controls with matched medications and comorbidities. Power spectra in delta to gamma frequency bands were compared during both REM and NREM sleep, between phasic and tonic REM sleep, and between the first versus last cycle of NREM sleep. Controls, but not RBD patients, displayed a decrease in beta power during phasic compared to tonic REM sleep. Compared to controls, RBD patients displayed a reduced decline in SWA from early to late NREM sleep. Overnight changes in the distribution of the amplitude of slow waves were also reduced in RBD patients. Without suppression of beta rhythms during phasic REM sleep, RBD patients might demonstrate heightened cortical arousal, favoring the emergence of behavioral episodes. A blunted difference between REM sleep sub-stages may constitute a sensitive biomarker for RBD. Moreover, reduced overnight decline in SWA suggests a reduced capacity for synaptic plasticity in RBD patients, which may favor progression towards neurodegenerative diseases.

## Introduction

Rapid eye movement (REM) sleep behavior disorder (RBD) is a parasomnia characterized by REM sleep abnormalities including failure to maintain muscle atonia resulting in nocturnal enactments of motor behaviors^1^. Several studies have shown that the presence of RBD also predicts the later occurrence of alpha-synucleinopathies such as Parkinson disease (PD), dementia with Lewy bodies (DLB), and multiple system atrophy (MSA). These findings suggest that RBD represents the initial manifestation of a variety of neurodegenerative processes^2–6^.

RBD patients display simple to elaborated, sometimes violent, motor behaviors during REM sleep, often associated with vivid dreaming and preferentially arising from phasic REM sleep^7–9^. In contrast to tonic REM sleep, phasic REM sleep is characterized by the presence of rapid eye movements (REMs), myoclonic twitches and irregularities in the vegetative system, paradoxically accompanied by an increase in arousal threshold^10^. Phasic REM sleep also differs from tonic REM sleep in terms of underlying neuronal activity^11,12^, sensory stimuli processing^11,13,14^, connectivity^15^, spontaneous oscillatory activity^16–18^ and dream recall^19–21^. More specifically, recent intracranial studies performed in epileptic patients revealed an electroencephalographic pattern of activation during phasic REM sleep in the motor cortex—decreased beta activity^22^—and amygdala—increased gamma activity^23^. In addition, several lines of evidence using fMRI/EEG/MEG also suggest the involvement of the visual system in relation to REMs^12,24,25^. If the regulation of phasic vs. tonic REM sleep was altered in motor, visual and limbic cortex in RBD patients, it may predispose them to behavioral enactment during REM sleep^26^. To date, how brain activity differs between phasic and tonic REM sleep in RBD patients however remains unclear, despite a first exploration by Sunwoo et al.^27^.

Cross-sectional studies have shown that cognitive performance is impaired in RBD patients across several domains: memory, executive functions and visuospatial abilities^28–33^. Up to 50% of RBD patients show mild cognitive impairment (MCI)^34^, suggesting an already altered function of several brain networks. Recent evidence suggest that the locus coeruleus may be altered in RBD patients^35–39^. Because the noradrenergic system plays a crucial role to allow synaptic potentiation during wake and subsequent synaptic homeostasis during sleep^40,41^, decreased noradrenergic tone in RBD patients may decrease the strength of their brain plasticity processes. Recent data suggest that sleep slow-wave activity (SWA) is tightly regulated by the amount of plasticity occurring during preceding wake and that sleep represents a sensitive window to investigate plastic processes taking place in the human brain. In line with the synaptic homeostasis hypothesis^42,43^, a wealth of data have indeed shown that cortical circuits undergo net synaptic potentiation during wake, and a subsequent renormalization of synaptic strength during sleep at night, which can both be tracked by assessing changes in EEG SWA during NREM sleep^44,45^. In short, SWA during NREM sleep is a marker of synaptic strength, and its overnight decline tracks synaptic downscaling, which is proportional to the strength of plasticity during wake and is thought to explain the beneficial cognitive effects of sleep. SWA during NREM sleep is thus sensitive to measure both the strength of structural connectivity and the strength of ongoing plasticity processes^46^. In addition to SWA, NREM sleep slow-wave parameters such as amplitude and slope are other indicators of synaptic strength^47^ that have not yet been examined in RBD patients. In the present work, we hypothesized that RBD patients may display not only REM sleep abnormalities, but also abnormal sleep SWA homeostasis during NREM sleep.

## Material and methods

### Subjects

Nine adult patients with a diagnosis of RBD and an equal number of matched non-medicated healthy controls (NMC) and of medicated controls (MC) without RBD were included in the analysis. All study procedures were reviewed and approved by the University of Wisconsin Health Sciences Institutional Review Board. All experiments were then performed in accordance with the relevant guidelines and regulations. All RBD patients and controls provided written informed consent before the procedures.

#### RBD patients

Patients were retrospectively identified from a patient cohort who had undergone overnight polysomnography (PSG) testing with combined hdEEG (hdPSG) at the Wisconsin Sleep Laboratory between 2008 and 2016. We selected patients referred to extended sleep EEG monitoring at the Wisconsin Sleep Clinics meeting the diagnostic criteria for RBD according to ICSD-3^48^ and evaluated by a Sleep Medicine board-certified physician (Dr. Ruth Benca). We excluded one RBD patient with concomitant epilepsy. Out of the nine remaining patients, seven were diagnosed with idiopathic RBD, while anti-depressant-related RBD could not be ruled out in two patients. Table 1 presents a summary of the clinical features of the RBD patients. Most were medicated for either comorbid psychiatric disorders such as depression and anxiety, or sleep disorders such as obstructive sleep apnea and periodic leg movement disorder. At the time of the study, patients had been experiencing motor enactments during sleep from between 6 months up to 35 years, with some no longer experiencing episodes due to treatment, and some still having episodes up to a weekly basis (Table 1). 8 out of 9 RBD patients consisted of early-onset RBD patients with first symptoms appearing before or at the age of 50.

**Table 1:** Medical details of the participants.

Group	Age (years)	Sex	Medication	Psychiatric disorders	Duration of RBD illness (years)	Frequency of RBD episodes
Non medicated controls	44	F	–	–	–	–
	46	F	–	–	–	–
	61	F	–	–	–	–
	34	M	–	–	–	–
	40	M	–	–	–	–
	48	M	–	–	–	–
	55	M	–	–	–	–
	60	M	–	–	–	–
	64	M	–	–	–	–
Medicated controls	28	F	Fluoxetine	MDD	–	–
	42	F	Buproprion, escitalopram	MDD	–	–
	60	F	–	–	–	–
	38	M	–	–	–	–
	43	M	Mirtazapine	–	–	–
	47	M	Lorazepam, mirtazapine, lamotrigine, 5 HTP	MDD, GAD	–	–
	50	M	Clonazepam, zolpidem, depakote	MDD, BP	–	–
	58	M	Venlaflaxine	MDD	–	–
	60	M	Alprazolam, zolpidem, olanzapine	MDD	–	–
RBD patients	43	F	Paroxetine, melatonin, tizanidine	MDD	18	n/a
	44	F	Clonazepam, pramipexole, topamax, gabapentin	–	3	Currently none
	61	F	Citalopram, alprazolam, clonazepam	MDD	35	Weekly
	36	M	–	MDD, GAD	2	n/a
	36	M	Citalopram, clonazepam	MDD	0.5	Monthly
	47	M	Buproprion, venlafaxine, clonazepam	MDD	2	Weekly
	55	M	Sertraline, clonazepam*	GAD	6	Monthly
	59	M	Clonazepam*, pramipexole	–	3	n/a
	66	M	–	–	16	Weekly

The duration of illness for RBD patients began when symptoms were first noticed. The frequency of episodes ranged from a few times per week to a few times per month. One participant on clonazepam was not having episodes at the time of their study. n/a indicates that the frequency could not be precisely determined because of an absence of a bed partner or because of a vague medical description (such as “chronically acts out dreams”).

*Indicates that clonazepam was tapered before the sleep study. The list of indicated medication includes any antidepressants, benzodiazepines, anticonvulsants or other CNS drugs, and excludes opioids, anti-hypertensives, corticosteroids, asthma medication or hormones. 5 HTP, serotonin; BP, bipolar disorder; GAD, general anxiety disorder; MDD, major depressive disorder. The medicated control group and RBD patients were not statistically different with regard to the occurrence of GAD, MDD, and the use of antidepressants, benzodiazepines, anticonvulsants and non-benzodiazepine hypnotics (Fisher’s exact tests: all *p* > 0.05).

#### Controls

Because most of our RBD patients had comorbid psychiatric and sleep disorders and were using medications that could affect their sleep, we used two groups of controls in our analysis (Table 1).

Age- and sex- matched, non-medicated controls (NMC) were drawn from a pool of subjects who participated in a study on the effects of meditation^49^. Exclusion criteria were (1) any current or past neuropsychiatric condition; (2) use of any psychotropic medication or medication that could affect sleep; and (3) evidence of any sleep disorder.

Age- and sex-matched, medicated controls (MC) were selected from patients who had undergone hdPSG at the Wisconsin Sleep Laboratory between 2008 and 2016 and had similar co-morbid disorders (such as major depressive disorder or general anxiety disorder) and medications (antidepressants, benzodiazepines or other CNS drugs) as our RBD cohort (see Table 1). The medicated control group and RBD patients were not statistically different with regard to the occurrence of major depressive disorder or generalized anxiety disorder, or the use of antidepressants, benzodiazepines, anticonvulsants and non-benzodiazepine hypnotics (Fisher’s exact tests: all *p* > 0.05).

### Sleep recordings

All participants underwent an overnight in-laboratory hdEEG recording (256 channels; Electrical Geodesics Inc., Eugene, OR; sampling rate 500 Hz) coupled with standard monitoring with electrooculogram (EOG), submental EMG, ECG, bilateral tibial EMG, respiratory inductance plethysmography, pulse oximetry and a position sensor. Timings of recordings were based on participants’ typical sleep schedule. Lights were switched off within 1 h of their usual bedtime.

Sleep staging was performed by a registered polysomnographic technician in 30-s epochs according to standard criteria^50^ using Alice Sleepware (Philips Respironics, Murrysville, PA) based on EOG, submental EMG and 6 hdEEG channels at the approximate 10–20 locations (F3, F4, C3, C4, O1, O2) re-referenced to the mastoids. All staging and scoring were reviewed by a board-certified sleep physician.

### Data analysis

#### EEG preprocessing

EEG signals were high-pass filtered at 0.1 Hz then down-sampled to 200 Hz and band-pass filtered (2-way least squares FIR, 1–40 Hz) in MATLAB (The MathWorks Inc., Natick, MA) using the EEGLAB toolbox^51^. Epochs of steady stage of NREM stage 2 and 3 (N2 and N3) sleep and REM sleep were extracted. From the REM epochs, tonic and phasic segments were manually defined by visual inspection for the presence of rapid eye movements (with ocular quiescence for 3 s defining tonic REM sleep). NREM sleep cycles were defined according to the modified criteria of Feinberg and Floyd^52,53^. To increase signal-to-noise ratio, analyses were then restricted to 173 channels overlaying the scalp, excluding channels from the face and the neck (channels falling within a plotting radius of 0.57 from the center of the head specified in the *topoplot* function of EEGLAB).

For REM sleep epochs, periods contaminated by artifacts were manually identified and rejected. For NREM sleep epochs, semi-automatic artifact rejection procedures were used to remove channels and epochs with high frequency noise, as previously done in^54–56^. Specifically, thresholds were automatically calculated for low (1–4 Hz) and high (20–40 Hz) frequency ranges at the 99th percentile for each channel. Spectral power in these ranges across all 6-s NREM epochs for each channel was plotted, visually inspected and used to remove epochs. For both NREM sleep and REM sleep, channels with artifacts affecting a majority of the recording were removed. Additional spectral-based and topographic procedures were used to remove individual channels with distinctly greater power relative to neighboring channels. Overall, less than 25% of the channels were removed and subsequently interpolated using spherical interpolation. As in previous studies, independent component analysis (ICA) implemented in the EEGLAB toolbox (*runica* algorithm) was then applied to remove physiological noise such as eye movements and heartbeats^54^, treating NREM and REM sleep and each patient separately. After component rejection, the signals were then re-referenced to the average of all channels.

#### Spectral analyses

For both REM sleep and NREM sleep, spectral analysis was performed using a Fourier transform on all clean 2-s epochs (Welch averaged modified periodogram with a Hamming window). Global power spectral densities (PSD) from 1 to 40 Hz, averaged across all channels were computed for all three groups for all-night N2/N3 sleep, all-night REM sleep, phasic REM sleep and tonic REM sleep. Topographic maps of absolute power in specific frequency bands (delta (1–4 Hz), theta (4–8 Hz), alpha (8–12 Hz), sigma (12–15 Hz), beta (15–25 Hz) and gamma (25–40 Hz)) were also computed in each group for all-night N2/N3 sleep, all-night REM sleep, first and last cycle of NREM sleep, phasic REM sleep and tonic REM sleep. In NREM sleep, delta band power is referred to as SWA.

#### Slow wave detection

Preprocessed EEG data were re-referenced to linked mastoids for a finer analysis of slow-wave characteristics. An automated detection algorithm based on zero crossings was used to identify individual slow waves^47,57^. Specifically, a negative-going signal envelope was calculated by selecting the fifth most negative sample across all selected channels (191 electrodes defined with a radius of 0.65 from the center of the head). Slow waves were defined as waves with frequency 0.5–2 Hz and no amplitude threshold. Slow-wave mean negative amplitude (μV) and negative slope (from the first zero-crossing to the negative peak) were estimated.

#### Statistical analyses

Between-group differences in demographic and polysomnographic variables (as shown in Table 2) were evaluated with one-way ANOVA followed by unpaired *t*-tests. Differences in PSDs between REM sleep sub-stages were tested with paired *t*-tests followed by false-discovery rate (FDR) correction. Differences in PSDs between the three groups during all-night REM and NREM sleep were tested using one-way ANOVA followed by FDR correction.

**Table 2:** PSG and demographic measures.

	Non medicated controls	Medicated controls	RBD patients	F(2,24) value	ANOVA *p* value
Age	46.8 ± 12.5	47.4 ± 11	49.7 ± 11	0.155	0.858
Sex (F/M)	3/6	3/6	3/6		N/A
TST (min)	365.7 ± 70.8	396.6 ± 75.7	359.6 ± 74.7	0.649	0.532
WASO (min)	75.0 ± 47.1	89.8 ± 36.2	128.4 ± 58.5	2.957	0.071
SOL (min)	18.2 ± 35.6	37.9 ± 32.3	26.2 ± 25.2	0.902	0.419
REML (min)	91.4 ± 30.1	174.8 ± 91.7	183.1 ± 108.0	3.311	0.053
SE (%)	80.0 ± 15.0	75.9 ± 9.9	70.4 ± 13.0	1.215	0.314
N1 (%)	4.5 ± 2.3*^	12.8 ± 8.4^	8.2 ± 4.4 *	4.874	**0.017**
N2 (%)	58.2 ± 9.5	63.4 ± 12.9	64.0 ± 12.3	0.686	0.513
N3 (%)	14.9 ± 8.9^	4.8 ± 4.4^	8.6 ± 9.7	3.609	**0.043**
REM (%)	22.4 ± 3.8	19.1 ± 9.4	19.2 ± 8.1	0.588	0.563
Tonic REM (%)	84.0 ± 7.2	76.3 ± 11.8	73.1 ± 11.8	1.934	0.10
Phasic REM (%)	16.0 ± 7.2	23.7 ± 11.8	26.9 ± 11.8	1.934	0.10
ArI(#/h)	12.7 ± 4.6	15.5 ± 7.9	15.8 ± 4.2	0.778	0.471
AHI (#/h)	5.8 ± 5.85	8.2 ± 6.3	6.1 ± 6.7	0.364	0.699
PLMI (#/h)	1.3 ± 0.59	1.06 ± 1.0	0.6 ± 1.0	1.427	0.260

Mean values (± standard deviation). Percentage values for sleep stages are expressed per total sleep time (TST).

*AHI* apnea–hypopnea index, *ArI* Arousal index, *PLMI* Periodic leg movement index, *SE* Sleep efficiency (TST per time in bed), *REML* Rapid eye movement onset latency, *SOL* Sleep onset latency, *WASO* Wake after sleep onset.

^ and *indicate significant unpaired t-test at α < 0.05 between non-medicated and medicated controls, and between non-medicated controls and RBD patients respectively. Significant *p* values for the main ANOVA test are indicated in bold.

**Figure 1:**
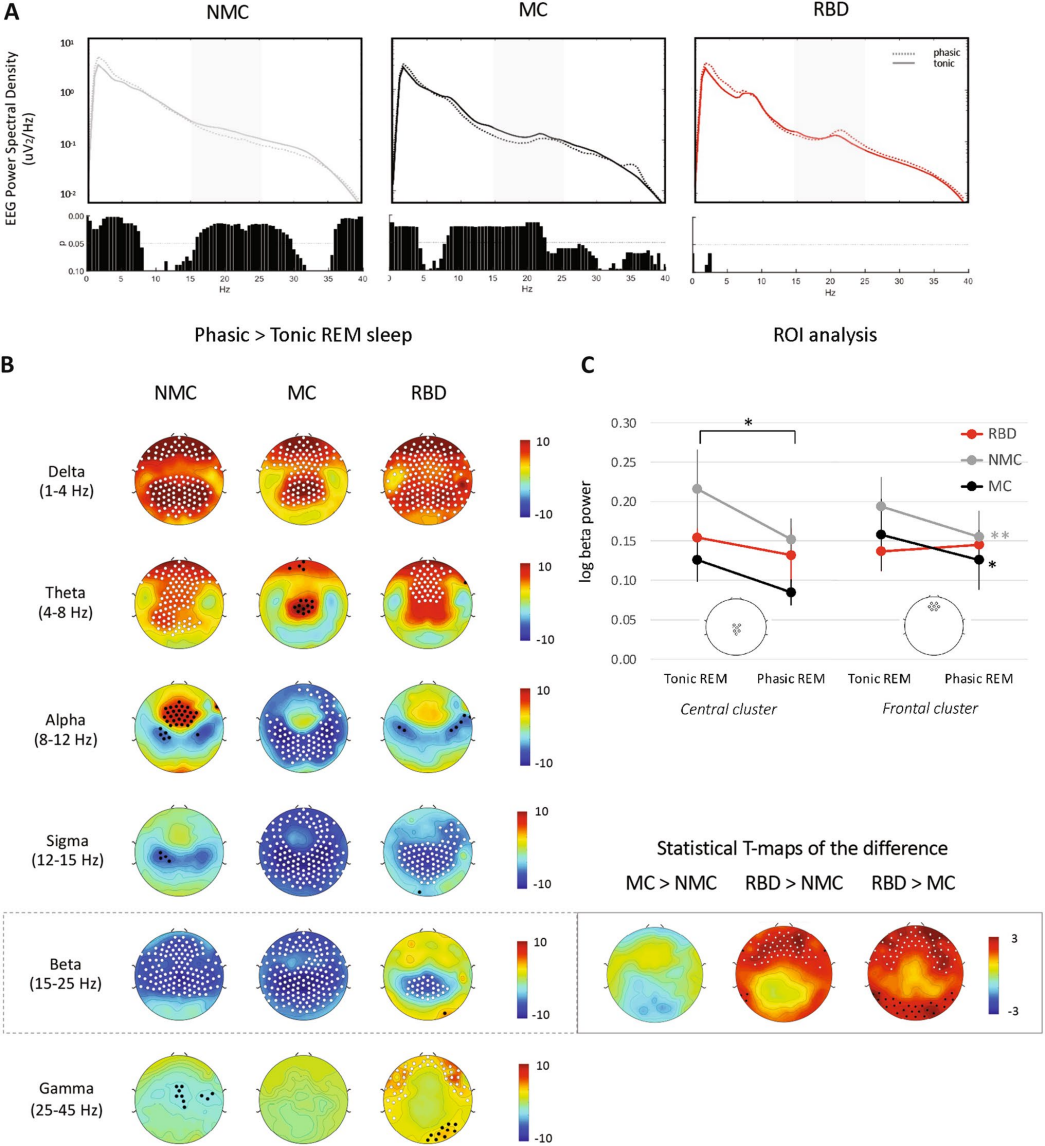
Spectral differences between phasic and tonic REM in RBD patients and controls. (**A**) Global EEG power spectra in phasic (dotted light) and tonic (continuous line) REM in the three groups. FDR-corrected *p* values from paired t-tests for the comparison between states within groups are shown below each plot, respectively. Greyed areas represent the beta band. (**B**) Normalized change in log power between phasic and tonic REM sleep across indicated frequency bands (delta: 1–4 Hz; theta: 4–8 Hz; alpha: 8–12 Hz; sigma: 12–15 Hz; beta: 15–25 Hz; and gamma: 25–40 Hz) for non-medicated controls (left column), medicated controls (middle column) and RBD patients (right column). White dots indicate *p* < 0.05 and black dots *p* < 0.1 after SNPM correction for one-sample t-test between both REM sleep sub-stages. Statistics on the changes showed significant differences between groups in the beta range only and are shown in the right insert. Individual channel t-maps are represented, with white dots indicating channels with SNPM corrected *p* < 0.05 and black dots uncorrected *p* < 0.05 after unpaired t-tests on the phasic to tonic REM power difference. (**C**) ROI analysis for central and frontal changes in absolute beta power between tonic and phasic REM sleep. Central and frontal ROIs were centered around Fz and Cz respectively and consisted of six and seven electrodes each (see inserts). Repeated-measure ANOVA with “group” and “REM sub-stage” on the log power revealed for the central cluster: a significant effect of REM sub-stage only (*p* < 0.01); for the frontal cluster: a significant effect of REM sub-stage (*p* < 0.01) and a trend for an interaction “group” × “REM sub-stage” (*p* = 0.056). * and ** for *p* < 0.05 and *p* < 0.01 Tukey Kramer post-hoc test between “tonic” and “phasic”.

**Figure 2:**
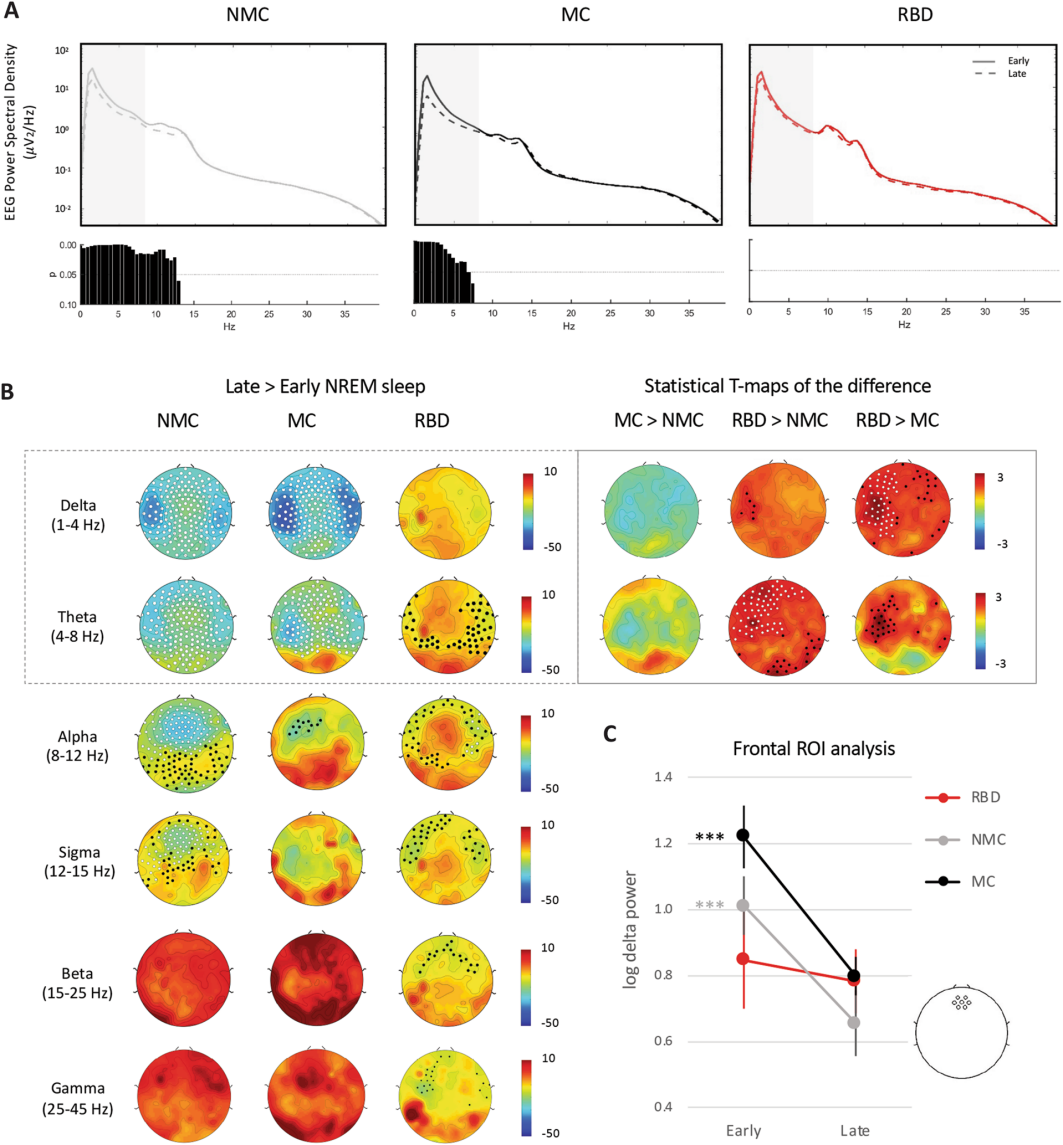
Delta and theta power as NREM sleep homeostasis markers in RBD patients and controls. (**A**) Global EEG power spectra in NREM sleep from the first cycle (continuous line) and last cycle (dashed line) in the three groups. FDR-corrected P values from paired t-tests for the comparison between states within groups are shown below each plot, respectively. Greyed areas represent the delta/SWA and theta band. (**B**) Normalized change in log power between the last and first cycle of NREM sleep in delta/SWA (1–4 Hz) and theta (4–8 Hz) in non-medicated controls (left), medicated controls (middle) and patients (right). Left columns: White dots indicate *p* < 0.05 and black dots *p* < 0.1 after SNPM correction for one sample t-test on the change. Statistics on the changes showed significant differences between groups in the delta and theta range only and are shown in the right insert. Individual channel t-maps are represented, with white dots indicating channels with SNPM corrected *p* < 0.05 and black dots uncorrected *p* < 0.05 after unpaired t-tests on the late to early NREM power difference. (**C**) ROI analysis for change in frontal absolute delta power/SWA between early and late night. Frontal ROI was centered around Fz and consisted of seven electrodes (see insert). The expected decrease in absolute delta power/SWA is visible in both control groups but not in patients. Repeated-measure ANOVA with “group” and “time” revealed a significant interaction (*p* = 0.012). *** p < 0.001 Tukey Kramer post-hoc test between “early” and “late”. The frontal delta power during the first cycle in RBD patients appeared lower compared to medicated controls at a trend level (*p* = 0.06).

Absolute power in scalp topographies were compared between groups using unpaired 2-tailed *t*-tests (such as in Supplementary Figs. 1 and 2). Paired *t*-tests were performed on absolute power topographies between states (phasic vs tonic REM in Fig. 1, early vs late NREM in Fig. 2). Absolute power in defined scalp regions (frontal and central clusters, consisting of 7 and 6 electrodes centered around Fz and Cz respectively) were compared in Region of Interest (ROI) analyses, with comparison between groups and states. Repeated-measure ANOVAs with “group” and “state” on absolute ROI powers were performed. Normalized power differences between states (first versus last NREM sleep cycle, phasic versus tonic REM sleep sub-stages) were calculated as the difference in power log values normalized by the power log value in the reference state (first NREM cycle and tonic REM sleep, respectively). To quantify differences between groups, we performed unpaired 2-tailed *t*-tests on these normalized power changes between states (inserts on the right in Figs. 1B, 2B). To identify significant clusters of electrodes, we used statistical nonparametric mapping with supra-threshold cluster tests to correct for multiple comparisons^58^ with a cluster-forming threshold of t = 2.12, corresponding to an uncorrected α level of *p* < 0.05. In brief, topographic power maps are randomly shuffled between groups in all possible combinations (n = 48,620). The size of the largest cluster above the threshold for each reshuffling is then used to create a maximal cluster size distribution. The suprathreshold cluster P value is then determined by comparison of the actual cluster size against the maximal cluster size distribution. This cluster-based method takes into account the relevant information provided by neighboring electrodes.

Changes in the distribution of slow-wave amplitude and slope from the first to the last NREM sleep cycle were assessed using repeated-measure ANOVA with factors “time” (early—late) and “group” (RBD, NMC, MC), followed by Bonferroni-corrected post-hoc tests. For all analyses, results were considered statistically significant *at p-*values < *0.*05 corrected for multiple comparisons*.*

## Results

### Sleep architecture

Sleep parameters derived from the hdPSG recordings are summarized in Table 2. RBD patients did not differ significantly from both medicated and non-medicated controls in terms of sleep duration, sleep efficiency or sleep disorder indices (AHI, apnea–hypopnea index; AI, arousal index; PLMI, periodic leg movement index). Each group had similar amount of phasic and tonic REM sleep (22 ± 11% phasic REM). Relative to NMC, RBD patients and MC had increased time spent in N1 sleep and decreased time spent in N3 sleep (% N1: *p* = 0.017, 8.2 to 12.8% in MC and RBD vs 4.5% in NMC, % N3: *p* = 0.043, 4.8 to 8.6% in MC and RBD vs 14.9% in NMC). One participant in the medicated control group did not have enough clean phasic REM (< 3 min) to be included in the analyses.

### REM sleep abnormalities

In order to examine how RBD affected EEG power during REM sleep, we first looked at power spectral densities during REM sleep averaged over the whole night. We found no differences between groups (Supplementary Fig. 1A). There were also no group differences in the topographical scalp maps in any frequency bands (Supplementary Fig. 1B).

To perform a more detailed analysis of REM sleep sub-stages, we then separated tonic vs phasic REM sleep. In both control groups, PSDs revealed an increased absolute power in the delta range and a decreased power in the beta range in phasic compared to tonic REM sleep (Fig. 1A). However, no significant differences between phasic and tonic REM sleep were found in the RBD patient group (Fig. 1A).

Scalp topographies of the power changes between phasic and tonic REM sleep confirmed the presence of a difference between RBD patients and controls. An increased delta power in fronto-central regions in phasic compared to tonic REM sleep was observed in both control groups as well as in RBD patients (Fig. 1B). However, while both control groups displayed a global reduction in beta power in phasic compared to tonic REM sleep, this decrease was limited to midline parietal regions in RBD patients (Fig. 1B). A post-hoc ROI analysis indeed illustrates that absolute beta power decreases from phasic to tonic REM in all groups in a central cluster of electrodes (ANOVA “sub-stage” *p* < 0.01) (Fig. 1C left). Statistical T-maps on the beta power change (Fig. 1B, right insert) confirmed that while all groups reported a decline of beta power in central regions, the beta power change in frontal regions was different in RBD patients compared to both control groups (50–54 electrodes with corrected *p* < 0.05 represented as white dots). A ROI analysis conducted in a frontal cluster confirmed a decrease in beta power in phasic REM sleep in both control groups (Fig. 1C right), but not in the RBD patient group (ANOVA “group x sub-stage” *p* = 0.056).

### NREM sleep abnormalities

We first checked for EEG spectral abnormalities in RBD patients during NREM sleep averaged across the whole night. Inspection of PSDs revealed no differences between groups (Supplementary Fig. 2A). With respect to the topographical maps (Supplementary Fig. 2B), RBD patients displayed lower theta power over bilateral central regions compared to NMC. However, this effect was not replicated when comparing them to MC (which were themselves not significantly different from NMC).

To quantify sleep homeostasis, we compared EEG spectral activity during NREM sleep from the early part of the night (first cycle) and last part of the night (last cycle). In both control groups, delta and theta power reduced significantly from the early to the late part of NREM sleep (Fig. 2A,B). In RBD patients, there was no significant decline in delta or theta power from the first to last part of NREM sleep (Fig. 2A,B). Direct comparison between groups (Fig. 2B, right insert), revealed that patients demonstrated significantly less delta and theta power reduction over the course of the night in the left fronto-central and parietal regions as compared to both control groups.

Since frontal SWA is known to show the most reliable sleep homeostasis EEG effects at the between-subject level^59,60^, we also performed a post-hoc ROI analysis of SWA changes from early to late NREM cycle (Fig. 2C) within a frontal cluster of electrodes. We confirmed that, on average, frontal SWA significantly declined in both control groups, but not within the RBD patient group (repeated measure ANOVA “group” × “time” *p* = 0.012, post-hoc effect of time *p* < 0.001 in NMC and MC). This seemed to be mostly driven by lower SWA at the beginning of the night in RBD patients: there was a trend towards lower frontal SWA in patients compared to controls during the first cycle (post-hoc between-group comparison: *p* = 0.06 for RBD versus MC and *p* > 0.1 for RBD versus NMC), while mean SWA was similar in patients and controls at the end of the night (Fig. 2C).

**Figure 3:**
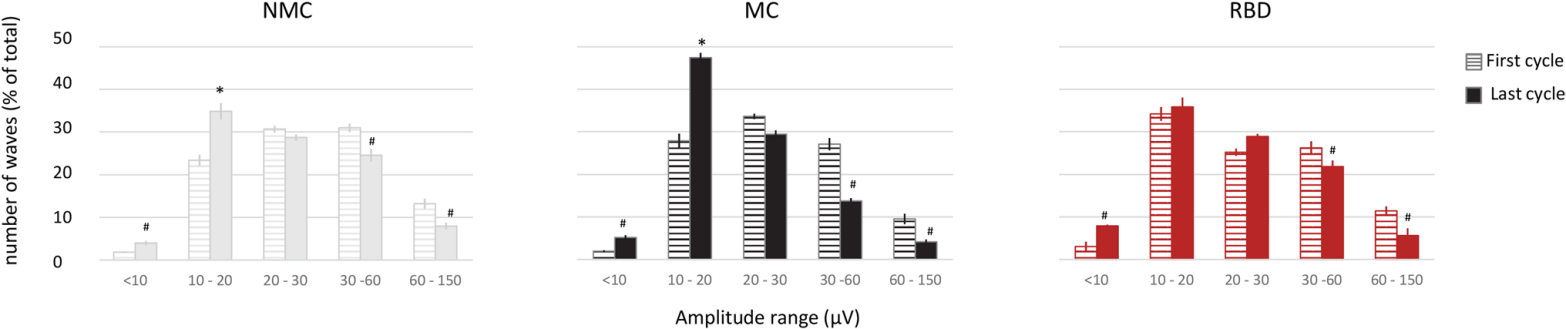
Distribution of the amplitude of slow waves during early and late NREM sleep. Proportion of slow waves in each amplitude range in early (first cycle, horizontal striped bars) and late sleep (last cycle, filled bars), in each group. Repeated-measure ANOVA with factors “time” (early—late) and “group” (RBD, NMC, MC) were performed for each amplitude bins. # indicate a significant (*p* < 0.01) main effect of “time” and Bonferroni-corrected post-hoc tests *indicate an effect of “time” only in control groups (“group” × “time” *p* = 0.021).

We also performed a detailed analysis of slow-wave characteristics. There was no significant effect of group on mean slow wave amplitude or slope changes from early to late sleep (Supplementary Table 1). However, when we analyzed the distribution of amplitude of slow wave between the beginning and end of the night, we again observed an abnormal pattern in RBD patients compared to controls. In both control groups, as expected^47^, slow waves with higher amplitude were more frequent during early sleep, and conversely, slow waves with lower amplitude were more frequent during later sleep (Fig. 3). In RBD patients, this shift was less pronounced, with decreased occurrence of low-amplitude, shallow slow waves during the second part of the night, especially for smaller waves in the 10–20 µV slow wave amplitude range (ANOVA *p* < 0.05 “group” × “time” interaction).

## Discussion

The present 256 electrode hdEEG study comparing nine RBD patients to two different control groups revealed consistent abnormalities in both REM sleep and NREM sleep. The decreased attenuation of beta frequency EEG activity during phasic compared to tonic REM sleep in RBD patients may reflect higher cortical arousal during phasic REM sleep, which may favor motor enactements. In addition, the reduced overnight decline in SWA observed in RBD patients during NREM sleep suggests a reduced capacity for neural plasticity in RBD patients, which may account for cognitive deficits and their predisposition to progress towards neurodegenerative diseases.

### REM sleep abnormalities

Our results indicate that contrasting brain activity during sub-stages of REM sleep may be helpful to understand the physiopathology of RBD. Indeed, no significant difference was found between RBD patients and controls when looking at overall REM sleep. These results are in line with the variable results found in the literature with two previous studies using standard low density PSG—EEG finding decreased beta power during REM sleep in RBD patients^61,62^, while others reported increases^63^ or no changes^27,64^. In contrast, our main REM sleep finding is that RBD patients showed a loss of differentiation in brain activity between phasic and tonic REM sleep. These results suggest that differences between phasic and tonic REM sleep may be a more sensitive diagnostic marker to detect RBD. To note, our finding of a loss of differentiation between REM sub-stages in RBD patients is limited to the beta band (15–20 Hz). This result should be interpreted with caution as this band has previously been shown to be affected by clonazepam, a medication taken by 4 of our 9 patients at the time of recording^64^. However, our analysis is controlled since we compared values within individuals between their two REM sub-stages and we showed no statistical differences between groups in absolute beta power in specific frontal and central regions. In addition, the chronic use of benzodiazepines is known to lead to less prominent sleep EEG changes^65^.

In healthy humans, phasic REM has been shown to be physiologically different from tonic REM sleep in several aspects. First, dreaming preferentially occurs in phasic REM periods^19–21^. In a human PET study, phasic REM was associated to primary occipital cortex activation (a region associated with visual mental imagery), while auditory evoked-related potentials^14^ and fMRI responses^11^ were suppressed in this sub-stage. In fact, the acoustic arousal threshold is higher in phasic REM sleep compared to tonic REM sleep^13^, and is overall highest compared to any other sleep stage. Previous studies also demonstrated a suppression of long-range inter- and intra- hemispheric EEG synchronization^15^, increased delta-theta activity^17,18^, increased gamma activity and decreased high alpha–beta activity in sensorimotor and higher order associative cortices^10,17,18,22^. With both our control groups, we here confirm the increase in delta/theta activity and attenuation of beta activity in phasic REM sleep. Our group recently showed that beta activity diffusely decreases during REM sleep compared to wake^66^, consistent with a decrease in cortical arousal. The dampened decrease in beta activity during phasic compared to tonic REM sleep in RBD patients suggests a reduction of the normal arousal suppression that protects active dreaming states. A central strength of our study is the use of high-density EEG—to our knowledge for the first time in patients with RBD—which allowed for a precise analysis of regional distribution of neural activity, suggesting that the abnormal regulation of REM sleep substages was most prominent within frontal cortices. Because frontal cortices are involved in motor behaviors, increased arousal in these regions during phasic REM sleep may predispose RBD patients to ‘act out their dreams’. Our results may at first appear to contrast with a study that found a larger suppression of beta activity in phasic REM sleep of RBD patients^27^. However, one should be cautious about the interpretation of the results. Sunwoo et al. had a moderate spatial resolution of 21 electrodes limiting the accurate separation of eye movements from brain signals using ICA, in contrast to our dense electrode array of 173 electrodes. In addition, their samples consisted of only 90 s of selected REM sleep period, in comparison with our use of the totality of non-artefactual REM sleep from the nocturnal recordings. Taken together, the characterization of EEG activity during tonic and phasic REM sleep in RBD patients warrants further investigation.

### NREM sleep abnormalities

RBD patients displayed a blunted overnight decrease in delta and theta power during NREM sleep, including a blunted decrease in slow-wave amplitude compared to both control groups. This dampened overnight decline seemed to be related to decreased frontal delta power at the beginning of the night (Fig. 2B), with not much change in power further happening over the course of the night. Overall these results may suggest reduced capacity for synaptic potentiation during wake in patients with RBD, which can be sensitively detected by the subsequent alteration in SWA homeostasis during NREM sleep. Sunwoo et al. very recently confirmed abnormalities in the morphology of NREM slow-waves in RBD patients, including decreased amplitude and slopes^67^.

Impairments of NREM sleep homeostasis and neuroplasticity have been observed in other patient populations, such as in MDD^68^. However, a pattern encompassing both REM and NREM sleep abnormalities seems unique to RBD patients and was not found in previous MDD literature nor in our MC controls. Future studies in larger groups of patients may clarify potentially differential but cumulative effects of RBD and MDD on NREM sleep homeostasis in medicated and non-medicated subjects.

The mechanisms of the neurodegenerative process in RBD remain unclear. The degeneration of the sublaterodorsal nucleus (SLD) in rodents—equivalent to the coeruleus/subcoeruleus nuclei complex in humans—is thought to be implicated in the loss of muscle atonia^69^. However, this pathology does not account for the wide variety of other non-motor symptoms observed in RBD. According to a current theory of RBD, the Braak’s ascending model, synuclein deposition in other brainstem nuclei may likely account for other RBD symptoms^70–72^. Of particular relevance to interpret the present findings is the evidence for a dysfunction in the central noradrenergic system in RBD patients—thought to be related to locus coeruleus damage. Indeed, brain imaging studies using neuromelanin-sensitive techniques demonstrated reduced signal intensity in the locus coeruleus/subcoeruleus complex in RBD patients^35,36^. Greater deposition of alpha-synuclein in the locus coeruleus (LC) were also found in autopsy specimens from RBD patients^37,38^. Another line of evidence suggesting impaired noradrenergic function in RBD comes from the observation that the amplitude of the event-related potential P300 during a visuospatial attention task—a marker of LC activity^73^—is reduced in RBD patients compared to controls^39^. Because the noradrenergic system plays a crucial role to allow synaptic potentiation during wake and subsequent synaptic homeostasis during sleep, decreased noradrenergic tone in RBD patients may decrease the strength of their brain plasticity processes. This would be in line with animal studies showing that pharmacological depletion of noradrenaline blunted SWA accumulation during wake and subsequent decrease during sleep in mice^40,41^.

Our findings may at first contrast with the results of a previous study performed on one central derivation (C3-A2) which found increased SWA during the first three sleep cycles in RBD patients compared to controls, with no differences on SWA changes over the course of the night^74^. However, increased SWA during NREM sleep in their RBD cohort could be accounted by the increased time they spent in N3 stage sleep—while in our study sleep parameters were not different between RBD patients and controls. Additionally, this prior investigation focused on absolute spectral power, and evaluated patients with a mean age nearly 20 years older than our current sample. Future larger studies that provide an ability to examine effects of age on EEG power spectra in RBD may further clarify the predictive power of absolute SWA versus overnight SWA changes as biomarkers predictive of cognitive impairment and of a future conversion to neurodegenerative disorders in RBD patients across mid-life to later adulthood.

### Limitations

One notable shortcoming of our study is its small sample size—which is due to both the retrospective nature of our work and the low prevalence of RBD (0.5–2% of the general population)^75–77^. However, our sample size is comparable to other published hdEEG sleep studies in patients with insomnia (n = 8)^56^, obstructive sleep apnea (n = 9)^54^ or NREM parasomnias (n = 15)^55^, in which controls were, like in our study, carefully age- and sex-matched.

Our analyses were not restricted to RBD subjects who were free of medications. As such, it more realistically reflects the nature of brain activity changes seen in RBD patients encountered in the sleep clinic. To take into account possible confounding factors, we both verified that the severity of comorbid sleep disorders (sleep apnea and period limb movement disorder indices) was similar between patients and controls and used a second MC group to account for the known effects of SSRIs^78^, benzodiazepines^79^ and depression^68^ on sleep homeostatic processes. Despite these efforts, our results may not necessarily be generalizable to all RBD patients. Future studies in larger clinical cohorts may assess the relative contribution of medication versus disease severity to EEG changes.

To note, two of our patients may have had anti-depressant related RBD. Current opinions in the field suggest that RBD with antidepressant can be considered as originating from the same underlying alpha-synucleinopathy-related neurodegeneration process rather than being a separate drug-induced condition^5,71^. Importantly, 8 out of our 9 patients consisted of “early-onset” RBD (onset of symptoms < or = to 50 years of age). This proportion is not anomalous, as in the recent years, it was noticed that RBD population at sleep centers consist of patients of younger age with a more equal representation of both sexes compared to typical RBD^80^. Early-onset RBD has been associated to a higher proportion of females, higher rates of depression and narcolepsy, and a lower rate of neurodegenerative disease such as PD, as opposed to late-onset RBD, more closely linked to elderly men and with a high probability for neurodegenerative diseases^81–83^. Age per se is also known to affect sleep SWA^84–86^. Our results should thus be interpreted with caution with respect to classical later-onset RBD, and larger cohort studies may in the future be able to assess a potential interaction between age, anti-depressant use and pathophysiologically distinct types of RBD.

Another potential caveat of this study is the single night recording design, which did not allow us to control for potential first-night effects^87,88^. To note, it was recently found that RBD patients are less likely to experience worse sleep during a first PSG night compared to other sleep-disordered patients^89^. Future studies may ideally replicate findings over the course of two nights.

Additionally, our cross-sectional design is not able to determine how our observed hdEEG alterations in REM and NREM sleep in RBD participants are related to risk of progression to symptomatic alpha-synucleinopathies. Thus, applying hdEEG to longitudinal studies of RBD may be a fruitful area of research, particularly in the future development of preventative strategies for these disorders.

Finally, we could not capture high quality wake data to compare to REM sleep and NREM sleep in our RBD patients. Such a comparison would be helpful to quantify within-subject differences in state-dependent SWA across these three states. Future prospective studies may aim to explicitly record a few minutes of full wakefulness before and after sleep in both controls and patients with RBD.

## Conclusion and future directions

Our hdEEG study revealed that our sample population of nine RBD patients, predominantly early-onset medicated individuals, display two clinically relevant alterations in brain activity during their sleep: (1) they showed an attenuated suppression of beta activity during phasic REM sleep compared to tonic REM sleep, which may predispose them to motor enactment during phasic REM sub-stage; (2) they showed a reduced overnight decline in SWA during NREM sleep, likely reflecting a decreased capacity for neural plasticity during wake. These findings were reproducible when comparing patients to both medicated and non-medicated controls that were matched for age and sex, and thus quite likely to be an RBD-specific effect.

Taken in the context of previous literature, our results may pave the way to further test the hypothesis that decreased noradrenergic tone during wake may lead to a reduced capacity for plastic changes in patients with RBD, which translates into a decrease in overnight SWA decline. Combined with REM abnormalities, this NREM sleep EEG profile could constitute a sensitive diagnostic biomarker for RBD that can be obtained from paraclinical studies.

## Supplementary Information

Supplementary Information

## Data Availability

The datasets generated during and/or analyzed during the current study are available from the corresponding author upon reasonable request.

## References

[1] Schenck CH, Bundlie SR, Ettinger MG, Mahowald MW (1986). Chronic behavioral disorders of human REM sleep: a new category of parasomnia. Sleep.

[2] Iranzo A (2014). Neurodegenerative disorder risk in idiopathic REM sleep behavior disorder: study in 174 patients. PLoS ONE.

[3] Schenck CH, Boeve BF, Mahowald MW (2013). Delayed emergence of a parkinsonian disorder or dementia in 81% of older men initially diagnosed with idiopathic rapid eye movement sleep behavior disorder: a 16-year update on a previously reported series. Sleep Med..

[4] Schenck CH, Bundlie SR, Mahowald MW (1996). Delayed emergence of a parkinsonian disorder in 38% of 29 older, men initially diagnosed with idiopathic rapid eye movement sleep behavior disorder. Neurology.

[5] Postuma RB, Gagnon JF, Montplaisir J (2013). Rapid eye movement sleep behavior disorder as a biomarker for neurodegeneration: the past 10 years. Sleep Med..

[6] Fernández-Arcos A, Iranzo A, Serradell M, Gaig C, Santamaria J (2016). The clinical phenotype of idiopathic rapid eye movement sleep behavior disorder at presentation: a study in 203 consecutive patients. Sleep.

[7] Arnulf I (2011). The ‘scanning hypothesis’ of rapid eye movements during REM sleep: a review of the evidence. Arch. Ital. Biol..

[8] Frauscher B (2009). The relation between abnormal behaviors and REM sleep microstructure in patients with REM sleep behavior disorder. Sleep Med..

[9] Manni R, Terzaghi M, Glorioso M (2009). Motor-behavioral episodes in REM sleep behavior disorder and phasic events during REM sleep. Sleep.

[10] Simor P, van der Wijk G, Nobili L, Peigneux P (2020). The microstructure of REM sleep: Why phasic and tonic?. Sleep Med. Rev..

[11] Wehrle R (2007). Functional microstates within human REM sleep: first evidence from fMRI of a thalamocortical network specific for phasic REM periods. Eur. J. Neurosci..

[12] Peigneux P (2001). Generation of rapid eye movements during paradoxical sleep in humans. Neuroimage.

[13] Ermis U, Krakow K, Voss U (2010). Arousal thresholds during human tonic and phasic REM sleep: phasic and tonic REM sleep. J. Sleep Res..

[14] Sallinen M, Kaartinen J, Lyytinen H (1996). Processing of auditory stimuli during tonic and phasic periods of REM sleep as revealed by event-related brain potentials. J. Sleep Res..

[15] Simor P, Gombos F, Blaskovich B, Bódizs R (2018). Long-range alpha and beta and short-range gamma EEG synchronization distinguishes phasic and tonic REM periods. Sleep.

[16] Simor P, Gombos F, Szakadát S, Sándor P, Bódizs R (2016). EEG spectral power in phasic and tonic REM sleep: Different patterns in young adults and children. J. Sleep Res..

[17] Simor P, van Der Wijk G, Gombos F, Kovács I (2019). The paradox of rapid eye movement sleep in the light of oscillatory activity and cortical synchronization during phasic and tonic microstates. Neuroimage.

[18] Jouny C, Chapotot F, Merica H (2000). EEG spectral activity during paradoxical sleep: further evidence for cognitive processing. NeuroReport.

[19] Pivik, R. T. Tonic states and phasic events in relation to sleep mentation. In *The mind in sleep: Psychology and psychophysiology. Wiley series on personality processes* (eds Ellman, S. J. & Antrobus J. S.) 214–247 (Wiley, 1991).

[20] Berger, R. J. & Oswald, I. Eye movements during active and passive dreams. *Science.* (1962). 10.1126/science.137.3530.601 10.1126/science.137.3530.601 13867678

[21] Stuart K, Conduit R (2009). Auditory inhibition of rapid eye movements and dream recall from REM sleep. Sleep.

[22] De Carli F (2016). Activation of the motor cortex during phasic rapid eye movement sleep. Ann. Neurol..

[23] Corsi-Cabrera M (2016). Human amygdala activation during rapid eye movements of rapid eye movement sleep: an intracranial study. J. Sleep Res..

[24] Miyauchi S, Misaki M, Kan S, Fukunaga T, Koike T (2009). Human brain activity time-locked to rapid eye movements during REM sleep. Exp. Brain Res..

[25] Ioannides AA (2004). MEG tomography of human cortex and brainstem activity in waking and REM sleep saccades. Cereb. Cortex.

[26] Proserpio, P., Terzaghi, M. & Nobili, L. Local cortical activations during REM sleep and implications for RBD. in *Rapid-eye-movement sleep behavior disorder* (2018). 10.1007/978-3-319-90152-7_29

[27] Sunwoo JS (2019). Abnormal activation of motor cortical network during phasic REM sleep in idiopathic REM sleep behavior disorder. Sleep.

[28] Gagnon JF (2009). Mild cognitive impairment in rapid eye movement sleep behavior disorder and Parkinson’s disease. Ann. Neurol..

[29] Gagnon JF, Bertrand JA, Marchand DG (2012). Cognition in rapid eye movement sleep behavior disorder. Front. Neurol..

[30] Terzaghi M (2008). Cognitive performance in REM sleep behaviour disorder: a possible early marker of neurodegenerative disease?. Sleep Med..

[31] Delazer M (2012). Decision making and executive functions in REM sleep behavior disorder. Sleep.

[32] Sasai T (2012). Impaired decision-making in idiopathic REM sleep behavior disorder. Sleep Med..

[33] Massicotte-Marquez J (2008). Executive dysfunction and memory impairment in idiopathic REM sleep behavior disorder. Neurology.

[34] Ferini-Strambi L, Fasiello E, Sforza M, Salsone M, Galbiati A (2019). Neuropsychological, electrophysiological, and neuroimaging biomarkers for REM behavior disorder. Expert Rev. Neurother..

[35] Ehrminger M (2016). The coeruleus/subcoeruleus complex in idiopathic rapid eye movement sleep behaviour disorder. Brain.

[36] Sommerauer M (2018). Evaluation of the noradrenergic system in Parkinson’s disease: an 11 C-MeNER PET and neuromelanin MRI study. Brain.

[37] Dugger BN (2012). Neuropathological analysis of brainstem cholinergic and catecholaminergic nuclei in relation to rapid eye movement (REM) sleep behaviour disorder. Neuropathol. Appl. Neurobiol..

[38] Postuma RB (2015). REM sleep behavior disorder and neuropathology in Parkinson’s disease. Mov. Disord..

[39] Byun JI (2017). Reduced P300 amplitude during a visuospatial attention task in idiopathic rapid eye movement sleep behavior disorder. Sleep Med..

[40] Cirelli C, Huber R, Gopalakrishnan A, Southard TL, Tononi G (2005). Locus ceruleus control of slow-wave homeostasis. J. Neurosci..

[41] Ouyang M, Hellman K, Abel T, Thomas SA (2004). Adrenergic signaling plays a critical role in the maintenance of waking and in the regulation of REM sleep. J. Neurophysiol..

[42] Tononi G, Cirelli C (2006). Sleep function and synaptic homeostasis. Sleep Med Rev.

[43] Tononi G, Cirelli C (2003). Sleep and synaptic homeostasis: a hypothesis. Brain Res Bull.

[44] Huber, R., Tononi, G. & Cirelli, C. Exploratory behavior, cortical BDNF expression, and sleep homeostasis. *Sleep* **2**, 129-139 10.1093/sleep/30.2.129 (2007). 10.1093/sleep/30.2.129 17326538

[45] Vyazovskiy VV, Cirelli C, Pfister-Genskow M, Faraguna U, Tononi G (2008). Molecular and electrophysiological evidence for net synaptic potentiation in wake and depression in sleep. Nat. Neurosci..

[46] Tononi G, Cirelli C (2014). Sleep and the price of plasticity: from synaptic and cellular homeostasis to memory consolidation and integration. Neuron.

[47] Riedner, B. A. *et al.* Sleep homeostasis and cortical synchronization: III. A high-density EEG study of sleep slow waves in humans. *Sleep* **30**, 1643–1657 (2007). 10.1093/sleep/30.12.1643 PMC2276133 18246974

[48] American Academy of Sleep Medicine. *International Classification of Sleep Disorders: Diagnostic and Coding Manual, 3nd edition*. *Diagnostic Coding Manual* (2014). 10.1111/febs.12678

[49] Ferrarelli, F. *et al.* Experienced Mindfulness Meditators Exhibit Higher Parietal-Occipital EEG Gamma Activity during NREM Sleep. *PLoS One* **8**, e73417 10.1371/journal.pone.0073417 (2013). 10.1371/journal.pone.0073417 PMC3756031 24015304

[50] Iber C, Ancoli-Israel S, Chesson A, Quan SF (2007). The AASM Manual for the Scoring of Sleep and Associated Events: Rules, Terminology, and Technical Specification.

[51] Delorme A, Makeig S (2004). EEGLAB: an open source toolbox for analysis of single-trial EEG dynamics including independent component analysis. J. Neurosci. Methods.

[52] Feinberg I, Floyd TC (1979). Systematic trends across the night in human sleep cycles. Psychophysiology.

[53] Aeschbach & Borbély (1993). All-night dynamics of the human sleep EEG. J. Sleep Res..

[54] Jones SG (2014). Regional reductions in sleep electroencephalography power in obstructive sleep apnea: a high-density EEG study. Sleep.

[55] Castelnovo A (2016). Scalp and source power topography in sleepwalking and sleep terrors: a high-density EEG study. Sleep.

[56] Riedner BA (2016). Regional patterns of elevated alpha and high-frequency electroencephalographic activity during nonrapid eye movement sleep in chronic insomnia: a pilot study. Sleep.

[57] Bernardi G, Siclari F, Handjaras G, Riedner BA, Tononi G (2018). Local and widespread slow waves in stable NREM sleep: evidence for distinct regulation mechanisms. Front. Hum. Neurosci..

[58] Nichols TE, Holmes AP (2002). Nonparametric permutation tests for functional neuroimaging: a primer with examples. Hum. Brain Mapp..

[59] Werth E, Achermann P, Borbély AA (1997). Fronto-occipital EEG power gradients in human sleep. J. Sleep Res..

[60] Cajochen C, Foy R, Dijk DJ (1999). Frontal predominance of a relative increase in sleep delta and theta EEG activity after sleep loss in humans. Sleep Res. Online.

[61] Fantini ML (2003). Slowing of electroencephalogram in rapid eye movement sleep behavior disorder. Ann. Neurol..

[62] Sasai T, Matsuura M, Inoue Y (2013). Electroencephalographic Findings Related With Mild Cognitive Impairment In Idiopathic Rapid Eye Movement Sleep Behavior Disorder. Sleep.

[63] Iranzo A (2010). Electroencephalographic slowing heralds mild cognitive impairment in idiopathic REM sleep behavior disorder. Sleep Med..

[64] Ferri, R. *et al.* REM sleep EEG instability in REM sleep behavior disorder and clonazepam effects. *Sleep* **40**(8), zsx080 10.1093/sleep/zsx080 (2017). 10.1093/sleep/zsx080 28482056

[65] Bastien CH, LeBlanc M, Carrier J, Morin CM (2003). Sleep EEG power spectra, insomnia, and chronic use of benzodiazepines. Sleep.

[66] Baird, B. *et al.* Human rapid eye movement sleep shows local increases in low-frequency oscillations and global decreases in high-frequency oscillations compared to resting wakefulness. *eNeuro* **5**, 1–11 (2018). 10.1523/ENEURO.0293-18.2018 PMC6140120 30225358

[67] Sunwoo J-S (2020). NREM sleep EEG oscillations in idiopathic REM sleep behavior disorder: a study of sleep spindles and slow oscillations. Sleep.

[68] Goldschmied, J. R. & Gehrman, P. An integrated model of slow-wave activity and neuroplasticity impairments in major depressive disorder. *Curr. Psychiatry Rep.* **21**(5), 30 10.1007/s11920-019-1013-4 (2019). 10.1007/s11920-019-1013-4 PMC6472258 30880367

[69] Garcia SV (2017). Genetic inactivation of glutamate neurons in the rat sublaterodorsal tegmental nucleus recapitulates REM sleep behaviour disorder. Brain.

[70] Heller J (2016). Brain imaging findings in idiopathic REM sleep behavior disorder (RBD)—a systematic review on potential biomarkers for neurodegeneration. Sleep Med. Rev..

[71] Högl B, Stefani A, Videnovic A (2018). Idiopathic REM sleep behaviour disorder and neurodegeneration—an update. Nat. Rev. Neurol..

[72] Boeve, B. F. REM sleep behavior disorder: updated review of the core features, the RBD-neurodegenerative disease association, evolving concepts, controversies, and future directions. *Ann. N. Y. Acad. Sci.* 15–54 (2010). 10.1111/j.1749-6632.2009.05115.x.REM 10.1111/j.1749-6632.2009.05115.x PMC2902006 20146689

[73] Nieuwenhuis S, Aston-Jones G, Cohen JD (2005). Decision making, the P3, and the locus coeruleus-norepinephrine system. Psychol. Bull..

[74] Massicotte-Marquez J (2005). Slow-wave sleep and delta power in rapid eye movement sleep behavior disorder. Ann. Neurol..

[75] Ohayon, M. M., Caulet, M. & Priest, R. G. Violent behavior during sleep. *J. Clin. Psychiatry* (1997). 9515980

[76] Kang S-H (2013). REM sleep behavior disorder in the korean elderly population: prevalence and clinical characteristics. Sleep.

[77] Haba-Rubio J (2018). Prevalence and determinants of rapid eye movement sleep behavior disorder in the general population. Sleep.

[78] Wichniak A, Wierzbicka A, Walęcka M, Jernajczyk W (2017). Effects of antidepressants on sleep. Curr. Psychiatry Rep..

[79] Borbely, A. A., Mattmann, P., Loepfe, M., Strauch, I. & Lehmann, D. Effect of benzodiazepine hypnotics on all-night sleep EEG spectra. *Hum. Neurobiol.* **4**, 189–194 (1985). 2866173

[80] Ju YE, Larson-Prior L, Duntley S (2011). Changing demographics in REM sleep behavior disorder: possible effect of autoimmunity and antidepressants. Sleep Med..

[81] Zhou J (2014). Characteristics of early- and late-onset rapid eye movement sleep behavior disorder in China: a case-control study. Sleep Med..

[82] Iranzo A (2005). Characteristics of idiopathic REM sleep behavior disorder and that associated with MSA and PD. Neurology.

[83] Chiaro G (2018). REM sleep behavior disorder, autonomic dysfunction and synuclein-related neurodegeneration: Where do we stand?. Clin. Auton. Res..

[84] Cajochen, C., Münch, M., Knoblauch, V., Blatter, K. & Wirz-Justice, A. Age-related changes in the circadian and homeostatic regulation of human sleep. In *Chronobiology International* (2006). 10.1080/07420520500545813 10.1080/07420520500545813 16687319

[85] Dijk DJ, Duffy JF, Czeisler CA (2000). Contribution of circadian physiology and sleep homeostasis to age-related changes in human sleep. Chronobiol. Int..

[86] Landolt HP, Dijk DJ, Achermann P, Borbély AA (1996). Effect of age on the sleep EEG: slow-wave activity and spindle frequency activity in young and middle-aged men. Brain Res..

[87] Tamaki M, Bang JW, Watanabe T, Sasaki Y (2016). Night watch in one brain hemisphere during sleep associated with the first-night effect in humans. Curr. Biol..

[88] Curcio G, Ferrara M, Piergianni A, Fratello F, De Gennaro L (2004). Paradoxes of the first-night effect: a quantitative analysis of antero-posterior EEG topography. Clin. Neurophysiol..

[89] Byun, J. H., Kim, K. T., Moon, H. J., Motamedi, G. K. & Cho, Y. W. The first night effect during polysomnography, and patients’ estimates of sleep quality. *Psychiatry Res.* **274**, 27–29 (2019). 10.1016/j.psychres.2019.02.011 30776709

