# Supplementary material for: A high-density electroencephalography study reveals abnormal sleep homeostasis in patients with rapid eye movement sleep behavior disorder
Source: Sci Rep. 2021 Feb 26;11:4758. doi: 10.1038/s41598-021-83980-w (PMC7910582; doi:10.1038/s41598-021-83980-w)
Supplement: Supplementary file 1 — Supplementary Information [file 41598_2021_83980_MOESM1_ESM.docx]

***A High-Density Electroencephalography Study Reveals Abnormal Sleep Homeostasis in Patients with Rapid Eye Movement Sleep Behavior Disorder***

Amandine Valomon ^1^, Brady A. Riedner ^1,2^, Stephanie G. Jones ^1,2^, Keith P. Nakamura ^3^, Giulio Tononi ^1^, David T. Plante ^1,2^ , Ruth M. Benca ^4^ , Melanie Boly ^1,3^

# Supplementary file

**Author affiliations:**

1 University of Wisconsin-Madison, Psychiatry - Wisconsin Institute for Sleep and Consciousness. Madison, WI, USA

2 University of Wisconsin-Madison School of Medicine and Public Health. Madison, WI, USA

3 University of Wisconsin-Madison, Neurology. Madison, WI, USA

4 University of California Irvine, Psychiatry and Human Behavior. Irvine, CA, USA

# Supplementary discussion

Medication effects

Most of our RBD patients and MC used CNS-medications such as SSRIs, anticonvulsants and benzodiazepines, and had occurrences of psychiatric disorders, such as depression and anxiety (Table 1). Numerous studies have shown the effect of depression, SSRIs and benzodiazepines, on SWS amount and REM sleep latency ^1–6^. In addition, depression has been shown to increase the frequency of REMs (Foster et al., 1976). Accordingly, we found in both RBD and MC groups a higher relative amount of N1 sleep, a reduced relative amount of N3 sleep and an increased REM sleep latency compared to the NMC group (effect of group ANOVA p=0.02, p=0.04, p=0.05 respectively, Table 2). However, there was no significant difference in RBD and MC groups compared to the NMC group concerning the percentage of phasic REM sleep (ANOVA p=0.10).

The RBD group seemed closer to the MC group and both differed from the NMC group on these sleep architecture observations, suggesting a possible effect of medication or co-morbid disorders for these measures. However, for our main topographical findings, the RBD group significantly differed from both the MC and NMC groups, suggesting an RBD-specific signature and not a medication effect.

Spindles

Spindles are generated through a complex relationship between the thalamus and cortical networks ^7^. Alterations in spindle generation have been proposed to correlate with various neurodegenerative disorders such as Parkinson’s disease ^8^. Two studies reported decreased spindle power in NREM sleep of RBD patients ^9,10^ in line with scarce reports of decreased spindle density in RBD ^11,12^. Our topographical data shows slightly reduced power in the spindle range during NREM sleep (Supp. Figure 2), although it does not reach significance, possibly due to low sample size and heterogeneity in disease duration or medication.

# Supplementary references

1. Plante DT, Landsness EC, Peterson MJ, et al. Sex-related differences in sleep slow wave activity in major depressive disorder: a high-density EEG investigation. *BMC Psychiatry*. 2012;12. doi:10.1186/1471-244X-12-146

2. Benca RM, Obermeyer WH, Thisted RA, Gillin JC. Sleep and Psychiatric Disorders: A Meta-analysis. *Arch Gen Psychiatry*. 1992. doi:10.1001/archpsyc.1992.01820080059010

3. Hoffmann R, Hendrickse W, Rush AJ, Armitage R. Slow-wave activity during non-REM sleep in men with schizophrenia and major depressive disorders. *Psychiatry Res*. 2000. doi:10.1016/S0165-1781(00)00181-5

4. Wichniak A, Wierzbicka A, Walęcka M, Jernajczyk W. Effects of Antidepressants on Sleep. *Curr Psychiatry Rep*. 2017;19(9):1-7. doi:10.1007/s11920-017-0816-4

5. Bastien CH, LeBlanc M, Carrier J, Morin CM. Sleep EEG power spectra, insomnia, and chronic use of benzodiazepines. *Sleep*. 2003. doi:10.1093/sleep/26.3.313

6. Borbely AA, Mattmann P, Loepfe M, Strauch I, Lehmann D. Effect of benzodiazepine hypnotics on all-night sleep EEG spectra. *Hum Neurobiol*. 1985.

7. Steriade M, Amzica F. Coalescence of sleep rhythms and their chronology in corticothalamic networks. *Sleep Res Online*. 1998.

8. Comella CL, Tanner CM, Ristanovic RK. Polysomnographic sleep measures in Parkinson’s disease patients with treatment‐induced hallucinations. *Ann Neurol*. 1993. doi:10.1002/ana.410340514

9. Ferri R, Rundo F, Silvani A, et al. REM Sleep EEG Instability in REM Sleep Behavior Disorder and Clonazepam Effects Raffaele. 2017;40(8).

10. Sunwoo J-S, Cha KS, Byun J-I, et al. NREM Sleep EEG Oscillations in Idiopathic REM Sleep Behavior Disorder: A study of sleep spindles and slow oscillations. *Sleep*. 2020. doi:10.1093/sleep/zsaa160

11. Christensen JAE, Kempfner J, Zoetmulder M, et al. Decreased sleep spindle density in patients with idiopathic REM sleep behavior disorder and patients with Parkinson’s disease. *Clin Neurophysiol*. 2014;125(3):512-519. doi:10.1016/j.clinph.2013.08.013

12. O’Reilly C, Godin I, Montplaisir J, Nielsen T. REM sleep behaviour disorder is associated with lower fast and higher slow sleep spindle densities. *J Sleep Res*. 2015;24(6):593-601. doi:10.1111/jsr.12309

# Supplementary Figures and Table

# Supplementary Figure 1. REM sleep alterations
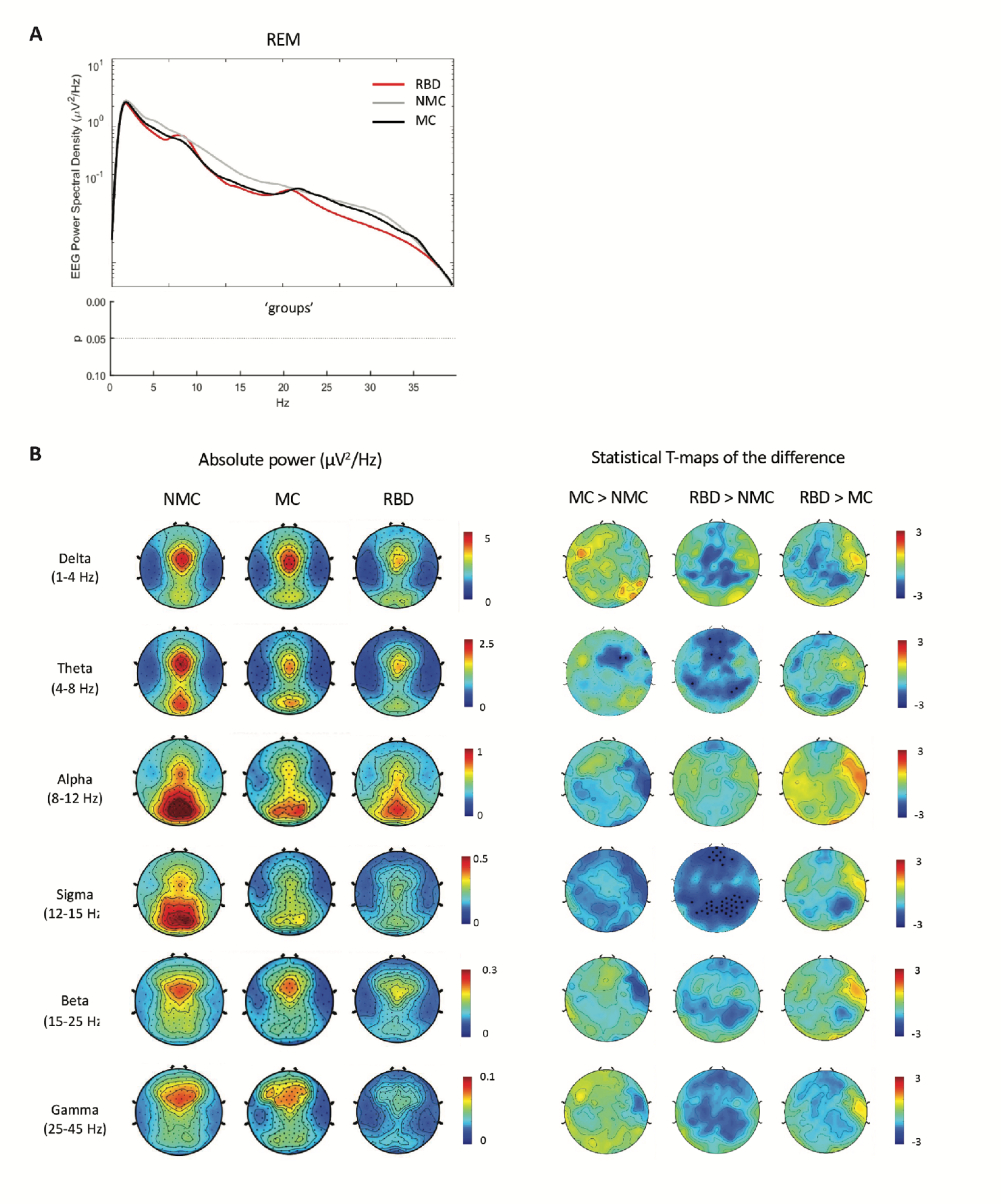


Average global absolute power in REM sleep (A) and topographies (B).

FDR corrected P values for one-way ANOVA on the logarithmic values of the global PSD revealed no significant differences between groups. Spectral power density averaged across indicated frequency bands (delta: 1–4 Hz; theta: 4–8 Hz; alpha: 8–12 Hz; sigma: 12–15 Hz; beta: 15–25 Hz; and gamma: 25–40 Hz) for NMC (left column), MC (middle column) and RBD patients (right column). Black dots are channels (total 173). Right columns show individual channel t-maps. White dots indicate channels with SNPM corrected P < 0.05 and black dots uncorrected P < 0.05 after unpaired t-tests.

# Supplementary Figure 2. NREM sleep alterations
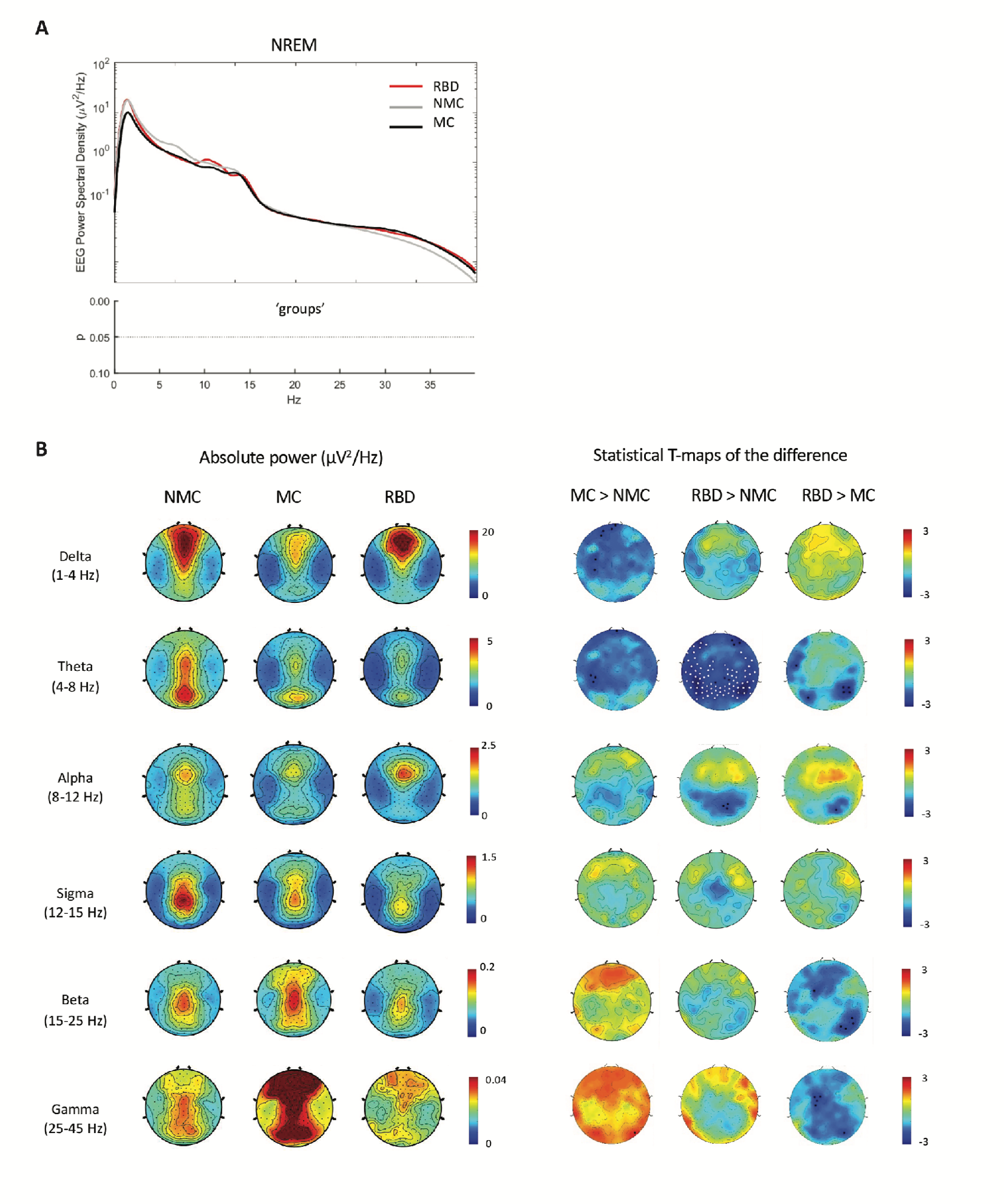


Average global absolute power in NREM sleep (A) and topographies (B).

FDR corrected P values for one-way ANOVA on the logarithmic values of the global PSD revealed no significant differences between groups. Spectral power density averaged across indicated frequency bands (delta: 1–4 Hz; theta: 4–8 Hz; alpha: 8–12 Hz; sigma: 12–15 Hz; beta: 15–25 Hz; and gamma: 25–40 Hz) for NMC (left column), MC (middle column) and RBD patients (right column). Black dots are channels (total 173). Right columns show individual channel t-maps. White dots indicate channels with SNPM corrected P < 0.05 and black dots uncorrected P < 0.05 after unpaired t-tests.

# Supplementary Table 1. Slow wave characteristics

Data are expressed as mean ± STD. Repeated measure ANOVA with group (NMC, MC and RBD patients) and time (early and late NREM sleep).
